# Tracking of leisure-time physical activity during adolescence and young adulthood: a 10-year longitudinal study

**DOI:** 10.1186/1479-5868-5-69

**Published:** 2008-12-29

**Authors:** Lise Kjønniksen, Torbjørn Torsheim, Bente Wold

**Affiliations:** 1 Faculty of Arts, Folk Culture and Teacher Education, Telemark University College, Norway; 2 Research Centre for Health Promotion, Faculty of Psychology, University of Bergen, Norway

## Abstract

**Background:**

The purpose of this study was to show how participation in leisure-time physical activity changes between ages 13 to 23, and to what extent engaging in specific types of sports tracks into young adulthood.

**Methods:**

The sample comprised 630 subjects who responded to questionnaires at age 13, with seven follow-ups over a 10-year period in the Norwegian Longitudinal Health Behaviour Study. The associations between adolescent participation in global and specific types of leisure-time physical activity were examined by analyses of variance, regression analysis and growth curve analysis.

**Results:**

The findings suggest that the transition from adolescence to adulthood is, on average, a period of decline in physical activity, but with the decline levelling off into adulthood. The decline was significantly greater among males than females. There were substantial individual differences in the amount of change, in particular among males.

Jogging alone and cycling, recreational activities such as skiing and hiking, and ball games, showed a high degree of tracking from age 15 to 23.

The findings indicate low associations between participation in specific types of activities during adolescence and global leisure-time physical activity in young adulthood, while participation in several adolescent physical activities simultaneously was moderately related to later activity. Thus, being involved in various types of physical activity may offer good opportunities for establishing lifelong involvement in physical activity, independent of the specific type of activity.

**Conclusion:**

The observed variation in change might suggest a need for a more targeted approach, with a focus on subgroups of individuals. The group of inactive youth may be considered as a high risk group, and the findings suggest that adolescent males who are inactive early seem likely to continue to be inactive later.

The observed heterogeneity in change highlights the limitation of previous approaches to analyzing physical levels over time, and suggests that multilevel analysis should be used in future research on longitudinal data on physical activity.

## Background

Adolescence has been described as a critical period during which involvement in physical activity might contribute to a physically active lifestyle lasting into adulthood [1,2]. The empirical evidence, however, is not conclusive. In their review, Trost et al. [3] concluded that there was sufficient evidence to upgrade the classification of activity history during childhood and youth and school sports from a repeatedly documented lack of association with adult activity to one of weak or mixed evidence of no association. The aim of the present study is to examine change and stability across global and specific indicators of leisure-time physical activity over a period of 10 years from age 13 to age 23.

Most longitudinal studies of physical activity from adolescence to adulthood have focused on tracking [4-6], usually defined as the relative maintenance of a variable's relative position in a group over time [7]. A number of recent long-term longitudinal studies report moderate to weak tracking coefficients [4-6,8-14]. In terms of describing the development of physical activity across time, a low tracking coefficient provides limited information. The low level of tracking might reflect at least two very different situations. The first situation could be labelled "temporal fluctuation". Even small fluctuations, if continued over time, would create a low level of tracking across the period, in particular when the period between measurements is long, and the reliability of indicators is imperfect.

A quite different situation which could contribute to low tracking is that of individual differences. Several studies have indicated an average decline in activity across age [4-6]. A low level of tracking might thus indicate that young people change at different rates across time. When individuals change at different rates, the relative order of individuals changes, leading to low tracking. Information about heterogeneity in change is essential in epidemiological studies, because such heterogeneity points to the need for both selected and universal approaches to the promotion of physical activity.

It has been suggested that tracking may be due to genetic factors or stable psychological characteristics of the individual such as psychological readiness for physical activity, or to stable conditions in the environment favouring physical activity such as high SES, or a family with high support for PA, as well as the learning of skills that may be more easily picked up at a later stage in life [8,15,16]. Involvement in youth sports usually implies engaging in frequent and repeated behaviour in a structured environmental context, offering children and adolescents experiences of repeated physical activity in stable circumstances. Thus involvement in youth sports is likely to be an important part of their life, representing a potentially strong influence on the formation of physical activity as a habit. [17]

Sallis et al. [18] suggested that one would expect higher tracking in activities that more easily transfer into adult activities, e.g. physical activities that can be performed outside a structured context. However, the most popular activities in adolescence are team sports such as football (soccer), usually implying the presence of a social structure such as a sports club. Telama et al. [19] argue that physical activity does not continue from youth to adulthood in the form of specific sports, in the sense that the types of activities adults participate in are usually different from common youth activities. The present theoretical and empirical understanding of the influence of various types of structured and unstructured physical activity during adolescence on adult physical activity is weak.

Participation in organized youth sports has been found to be consistently associated with a higher level of adult physical activity [6,13-15,19]. One of the few studies reporting the tracking of specific types of sports, and the effect of these on adult physical activity in general, is the 1966 birth cohort study from North Finland [15]. Adolescent participation in ball games at age 14 was associated with participation in ball games, as well as with general physical activity level, at age 31 in males. The same was the case for cross-country skiing and running. In females, adolescent participation in gymnastics and cycling was found to track into adulthood, while participation in ball games was not significantly related to later activity. The study may have several limitations, notably that it is difficult to assess whether these findings can be generalized to other populations. Types and organization of youth sports may vary between countries, and may also have changed for generations born later than 1966, suggesting a need for further studies.

With this background, the present study aims to examine change and stability in global and specific types of leisure-time physical activity during adolescence and young adulthood. More specifically, the research questions are:

1. To what extent do participation in global leisure time physical activity and recreational activities change during ages 13 to 23?

2. To what extent is changes in participation in global leisure time physical activity and recreational activity interrelated?

3. To what extent does baseline level of participation in global leisure time physical activity and recreational activity predict subsequent participation during ages 14 to 23?

4. To what extent does participation in specific types of leisure-time physical activity track from ages 15 to 23?

5. To what extent does participation in several specific types of leisure-time physical activity simultaneously at age 15 predict participation in global leisure-time physical activity at age 23?

6. To what extent does participation in specific types of leisure-time physical activity at age 15 predict global leisure-time physical activity at age 23?

## Methods

### Design and sample

The Norwegian Longitudinal Health Behaviour Study is both a longitudinal and a two-generational study. The same subjects were questioned from age 13 in 1990 until age 23 in 2000. The subjects were surveyed eight times (1990, 1991, 1992, 1993, 1994, 1995, 1998 and 2000).

The baseline sample consisted of students from 22 schools (54 classes) which were randomly selected by taking every 5th school from an alphabetic list of schools in Hordaland county in western Norway.

In the 1990 study, 924 students participated; there were 510 males (55%) and 414 females (45%). This was 77% of the initial sample of 1195 students who were invited to take part in the study. The average age was 13.3 years. The main reasons for non-response in the first survey were that parents did not return written consent for their children to participate (19%, n = 222), students who did not want to participate (4%, n = 46) and students who provided incomplete responses (0.3%, n = 3).

Table 1 shows the number of respondents in all surveys. In the final survey, 630 subjects responded. The measure of leisure-time physical activity (number of times doing sports or exercise per week) in this study was identical to the measure used with a nationally representative sample of 13-year-olds (n = 1616) in the 1989 Health Behaviour in School-aged Children study in Norway. The baseline mean of this variable in the present study was almost identical to that of the national sample (3.0 versus 3.2), suggesting that our sample is representative of Norwegian youth in terms of their physical activity.

**Table 1:** Number of respondents by age and year of data collection

Year	1990	1991	1992	1993	1995	1996	1998	2000
Age	13	14	15	16	18	19	21	23
N^1^	924	958	936	789	779	643	634	630

^1 ^During the first two years of the study those families and students who moved to the district of the participating schools were invited to enrol into the study, thus the n increased the first two years.

### Questionnaire

#### Global leisure-time physical activity

This item was adopted from the Health Behaviour in School-aged Children, a WHO cross-national survey (the HBSC study), where it has been used in seven large-scale international surveys since 1983 [20]. The item reads: "In your leisure time, how often do you do sports or exercise until you are out of breath or sweat?" Response keys (coding in the analyses in parenthesis) were every day (7), 4–6 times per week (5), 2–3 times per week (2.5), once per week (1), 1–3 times per month (0.5), less than once per month (0), and never (0). This item was included in all eight time points. Baseline status was divided into three groups. The low activity group consisted of individuals with less than weekly activity, the moderate activity group consisted of those being active one to three times a week, and the high activity group consisted of those being active four times or more weekly.

A test-retest study of this item among 15-year-old Norwegian students found the intraclass correlation (ICC) to be 0.74 which is considered highly stable. This item was also found to be acceptably reliable in an Australian study, with 70% agreement between two-way classifications of 13- and 15-year-olds (active/insufficiently active) after an interval of two weeks [21]. A similar single "sweat" question has been found to correlate well with maximal oxygen uptake [4,22,23]. In their study of 32 male twins (16 twin pairs aged 16, 17 and 18), Aarnio et al. [22] reported that a single self-report question about frequency of activity was more strongly related to maximal oxygen uptake (VO2max) than a more complicated index of activities.

#### Outdoor recreational activity

The measure consisted of two questions. These were: "How often do you usually do outdoor activity in summer?" and "How often do you usually do outdoor activity in winter?" Possible responses were, four times a week or more often, about 2–3 times a week, once a week, less than once a week, and never (the two questions were added together and averaged).

#### Specific types of physical activities

At ages 15 and 23, subjects were asked how often they had participated in various types of physical activities in their leisure time during the past year. A list of 31 different physical activity alternatives was included, with four response categories (several times a week, once a week, less than once a week, and never). In 2000, exercising in a fitness centre was added to the list.

### Procedures

All administrative levels in the formal school system were contacted via mail in spring 1990, and informed about the study. All levels above parents such as teachers and headmaster accepted the general goals of the study, and agreed to participate in the project. Parents were asked to give their written consent. At data collections in 1990, 1991 and 1992, the respondents were contacted at their schools, and the questionnaires were handed out by university staff during school hours, without teachers being present in class. From 1993 (when some respondents had left obligatory school at age 16), the questionnaire was sent by mail to the participants. Strict procedures were followed to ensure confidentiality, and the study was approved by the Norwegian Data Inspectorate. Informed consent and local ethics committee approval was obtained.

### Data analysis

SPSS version 14.0 (SPSS Inc., Chicago) was applied for analyses of bivariate correlations, Student's *t*-test, General Linear Model (GLM) for repeated measures, logistic regression analysis, stepwise regression analysis and McNemar's test. Growth curve analysis [24] was applied to describe changes in physical activity during time.

The aim of the first set of analyses was to examine multivariate change in global leisure-time physical activity and recreational activity, and to determine whether these different domains of activity showed a correlated level of change from age 13 to 23, or whether they showed independent trajectories.

To examine the extent of absolute stability from ages 13 to 23, a series of growth curve models was tested in which baseline status of leisure-time physical activity was used as a time-invariant covariate for growth in subsequent ages. To avoid confusion between baseline status of activity (age 13) and change, the dependent measures of activity only included observations from ages 14 to 23. If baseline status was maintained, one would expect parallel curves of development for the baseline groups. In contrast, if baseline status was not maintained, curves would be expected to regress towards the mean. Two models were compared. Model 1 included a common linear growth curve for all levels of baseline status, indicating that differences at baseline would remain constant across the period, irrespective of the overall pattern of declining activity. Model 2 included interaction terms between baseline status and subsequent change, thus allowing for regression towards the mean over time. According to the latter model, the rate of change would be different depending on baseline status, with decreasing effects of baseline across age.

To study overall stability in specific physical activities, we conducted a marginal analysis using McNemar's tests for correlated proportions. We also estimated conditional logistic regression models, regressing participation at age 23 on participation at age 15. Linear regression analysis was conducted to examine the associations between specific physical activities and global leisure-time physical activity.

## Results

### Drop-out analysis

Drop-out analysis was performed by comparing baseline values of physical activity and recreational activities, as well as participation in specific sports at the age of 15, between those who dropped out of the study before age 23 and those who stayed in the study. Thirty-five percent dropped out during the 10 years after the baseline survey. Significantly more males dropped out than females: 64% of the drop-outs were males and 36% were females (Chi-square = 19.68, *p *< .01). No differences were found between drop-outs and those staying in the study in the occupational status of their mothers and fathers, or in the respondents' reported plans for future education.

Participation in leisure-time physical activity measured by a global variable and two variables on recreational activity during summer and winter at age 13 did not differ significantly between those who dropped out and those who stayed in the study, except that females dropping out of the study had a statistically significantly higher baseline level of global leisure time physical activity (3.0 versus 2.5 times per week). Among females, there were no statistically significant differences in participation in specific sports at age 15 between drop-outs and those staying in the study, while participation in power sports and bodybuilding was somewhat higher among males who dropped out of the study compared with those who stayed on.

### Intra-individual change in recreational activity and physical activity from ages 13 to 23

The results of fitting a multivariate multilevel model of change are presented in Table 2a. It can be seen that there was an average decline in global leisure time physical activity and recreational activity for both males and females, indicated by a negative fixed linear effect of age centred at age 18. The positive quadratic effect indicates that the decrease across time was decremental across the period. Notably, the average decline in physical activity per year was stronger in males (B = -0.17) than in females (B = -0.09).

**Table 2:** 

**a. Multivariate model of change in global leisure-time physical and recreational activity from age 13 to 23 with linear (and quadratic) fixed effects**								
	Males		Females					
						
	B	SE	B	SE				
				
Physical activity	.	.	.	.				
Intercept	2.628	0.082	2.101	0.072				
Age-18.3	-0.1704	0.0127	-0.0945	0.0118				
(Age-18.3)^2^	0.006	0.003	0.013	0.003				
Recreational activity								
Intercept	2.436	0.058	2.472	0.057				
Age-18.3	-0.118	0.009	-0.090	0.0089				
(Age-18.3)2	0.010	0.003	0.014	0.002				

**b. Random variance components from multivariate model of change in global leisure-time physical and recreational activity from age 13 to 23 (correlations in plain style, covariances in bold).**								

	Males				Females			
		
.	1.	2.	3.	4.	1.	2.	3.	4.

Between persons								
1. Intercept recreational activity	**0.89**	.	.	.	**0.72**	.	.	.
2. Intercept physical activity	0.57	**1.86**	.	.	0.49	**1.05**	.	.
3. Slope recreational activity	0.01	0.42	**0.01**	.	0.03	0.24	**0.01**	.-
4. Slope physical activity	0.19	0.06	0.78	**0.02**	0.04	-0.16	0.30	**0.02**
Within persons								
1. Intercept recreational activity	**1.32**	-	-	**-**	**1.21**	-	-	**-**
2. Intercept physical activity	0.17	**2.49**	-	**-**	0.14	**2.06**	-	**-**

The random part of this analysis is shown in Table 2b. The table shows several notable results. First, independent of sex, the intercept covariance suggests that participation in global leisure-time physical activity at age 18 also indicates recreational activity at age 18. The table reveals sex differences. First, the estimated intercept variance among males was clearly stronger than that of females, suggesting that male levels of activity were more heterogeneous than those of females. Secondly, the covariance between change in physical activity and change in recreational activity was strong in males, but not in females. This means that males who tended to decrease their level of physical activity also tended to decrease their level of recreational activity. For females, changes in recreational activity and physical activity were only moderately correlated, indicating a stronger specificity in the amount of change across activity domains.

**Figure 1:**
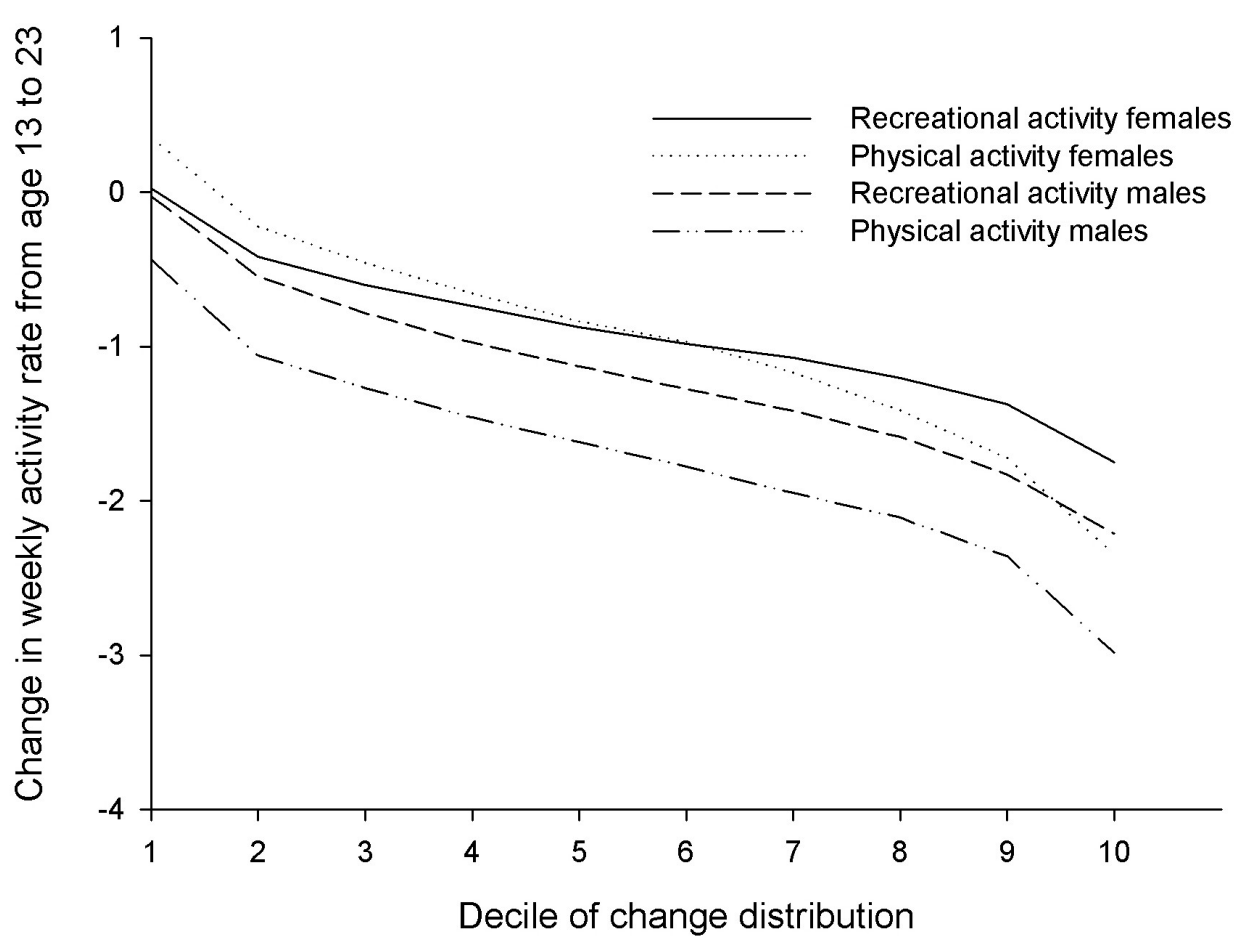
**Change in physical and recreational activity from age 13 to 23 by deciles of change distribution and sex**.

In order to interpret the amount of heterogeneity in change across time, the expected posterior slope of activity was computed for each individual and ranked. The deciles of the posterior levels of physical activity and the lowest and highest deciles of these ranks are shown in Figure 1. The figure shows several important individual differences in change. First, the entire distribution of slopes is located below zero, indicating a negative slope for all individuals. Second, the distribution for males was located more negatively, indicating a stronger decrease in physical activity than for females.

### Stability of baseline differences in global physical activity and recreational activity from ages 13 to 23

Table 3 shows the results of fitting a multilevel model of change from ages 14 to 23, conditional on activity status at age 13. Baseline-by-age interactions were statistically significant, indicating that the impact of baseline decreased across ages. A male reporting being active four times or more weekly at age 13 would be expected to be active 2.25 days a week as a 23-year-old. In contrast, a male reporting being inactive at age 13 would be expected to only be active 1.08 days a week as a 23-year-old. For females, a similar level of activity maintenance was evident. Females being active more than four times a week at age 13 would be predicted to be active 2.25 days a week at age 23. Females who were inactive at age 13 would be expected to be active an average of 1.72 times a week as a 23-year-old.

**Table 3:** Global leisure-time physical and recreational activity at age 13 as predictor for subsequent tracking and regression in subsequent corresponding activity from age 14 to age 23

	Predicted frequency of corresponding activity at age 23			
	
	Males		Females	
		
Activity status at age 13	B	95% CI	B	95% CI
Global leisure physical activity				
Inactive (< = 1 time a week)	1.08	(0.38,1.79)	1.72	(1.09, 2.35)
Regular activity (1–3 times a week)	1.68	(1.35, 2.01)	1.80	(1.56, 2.04)
High activity (> = 4 times a week)	2.25	(1.91, 2.59)	2.25	(1.80, 2.70)
Recreational activity				
Inactive (< = 1 time a week)	1.34	(0.63, 2.05)	2.36	(1.60, 3.12)
Regular activity (1–3 times a week)	2.03	(1.80, 2.25)	2.12	(1.90, 2.34)
High activity (> = 4 times a week)	2.09	(1.86, 2.33)	2.56	(2.34, 2.78)

A pattern of regression towards the mean was also evident for recreational activity (Table 3). Males reporting recreational activity four times or more weekly at age 13 had an average level of 2.09 days active days a week at age 23. Males who were inactive at baseline were on average active 1.34 days a week at age 23. For females there were even stronger changes. A female who was active four or more times weekly at baseline would be expected to be active 2.56 times a week at age 23. In contrast, a female who was inactive at age 13 would be expected to increase her level of recreational activity to 2.36 days per week.

### Stability of participation in sports

In both sexes, walking fast, cycling, and hiking were among the most frequent activities reported at age 15. Among males, playing soccer was the most frequently reported ball game, while females more frequently reported other ball games such as handball. As suggested by the McNemar's test of change in proportions, there was a considerable decline in the participation rates in almost all activities, with the exception of walking, power sports and hard work such as gardening. A question concerning exercise in fitness centres or gyms was included in the data collection among the 23-year-olds. Forty per cent of the females reported exercising once a week or more in a fitness centre at age 23, as compared with 23% of the males (Chi square 21.34, df = 3, *p *< .001).

There was a significant decline with regard to the number of specific activities the respondents reported from ages 15 to 23 (Table 4), and males reported being engaged in significantly more activities than girls at both ages. A significant interaction effect between time and sex was also found, with the decline in number of activities being greater among males.

**Table 4:** Mean number of specific types of leisure-time physical activity at ages 15 and 23 by sex

Sex	AGE 15		AGE 23			
	Mean	Stand. Dev.	Mean	Stand. Dev.	*t*	*p*
MALES (N = 210)	7.5	5.22	4.0	2.83	-9.86	***
FEMALES (N = 214)	5.7	3.49	3.5	2.33	-8.60	***
						
*t*-test sex diff.	4.09***		2.54*			

* *p *< .05, *** *p *< .001

The odds ratio of participating in a specific type of physical activity at age 23 conditional on participation status at age 15 is shown in Table 5. It can be seen that the conditional stability of participation differed considerably across types of sport. Among males, participation in 12 specific activities at age 15 was associated with increased odds of participation in the same activities at age 23, notably soccer and waterskiing, as well as cross-country and alpine skiing. Similarly, among females who played soccer, other ball games or did alpine skiing at age 15 there was an increase in odds to be involved in the same activities at age 23. The confidence intervals were rather wide, probably because the rate of participation in these activities was low among the 23 year-olds.

**Table 5:** Odds ratio of participation in specific physical activities at age 23 as predicted by participation in the same activities at age 15 with non-participation at age 15 as reference (only activities reported by at least 5% of the respondents at age 23 were included)

Males			Females		
	
Ranks of activity type	OR	95% CI	Ranks of activity type	OR	95% CI
1. Soccer	4.82	(2.78–8.38)	1. Ball games	7.73	(3.19–18.72)
2. Waterskiing	4.11	(2.38–7.09)	2. Alpine skiing, ski jump	6.25	(3.53–11.06)
3. Alpine skiing, ski jump	4.00	(2.17–7.37)	3. Soccer	6.04	(2.59–14.11)
4. Cross-country skiing	3.51	(1.99–6.19	4. Jogging alone	2.37	(1.30–4.30)
5. Judo, karate	3.13	(1.13–8.68)	5. Aerobics	2.20	(1.36–3.57)
6. Power sports	2.96	(1.78–4.92)	6. Swimming, diving	2.10	(1.27–3.46)
7. Swimming, diving	2.82	(1.44–4.95)	7. Walking, hiking	1.87	(0.39–8.93)
8. Ball games	2.69	(1.46–4.95)	8. Hard work	1.66	(0.82–3.34)
9. Cycling practice	2.31	(1.38–3.85)	9. Cycling > 10 min	1.63	(0.98–2.71)
10. Jogging with others	2.23	(1.22–4.08)	10. Dance	1.52	(0.93–2.50)
11. Jogging alone	2.20	(1.28–3.78)	11. Cycling practice	1.46	(0.88–2.41)
12. Bodybuilding	2.02	(1.08–3.79)	12. Power sports	1.41	(0.79–2.53)
13. Cycling > 10 min	1.89	(1.03–3.45)			
14. Hard work	1.23	(0.26–5.74)			
15. Walk fast > 10 min	0.88	(0.25–3.13)			
16. Walking, hiking	0.51	(0.12–2.24)			

### Association between specific activities in adolescence and adult physical activity level

The Pearson correlation between number of physical activities engaged in at age 15 and leisure-time physical activity at age 23 was 0.21 (*p *< .01) among males, and 0.23 among females (*p *< .001), suggesting a moderate association between breadth of involvement in adolescence and later activity.

Table 6 shows the results of the regression analyses of the association between specific types of physical activities at age 15 and level of global leisure-time physical activity at age 23. Among males, track and field running, playing soccer and walking fast for more than 10 minutes were found to be statistically significant. Among females, jogging with others, hiking and waterskiing in summer were positively and statistically significantly associated with global activity level at age 23 after controlling for all other activities. Swimming and diving during summer at age 15 was negatively associated with later global activity. However, participation in these activities at age 15 explained a relatively low proportion of the variance in global leisure-time physical activity at age 23.

**Table 6:** Participation in global leisure-time physical activity (times per week) at age 23 by participation in specific physical activities at age 15.

	Unstand. B	Stand. Beta	*t*	R ^2^
MALES				
Constant	4.48		9.26***	
Track & field running	0.43	0,21	3.27**	
Soccer	0.29	0,17	2.75*	
Walk fast > 10 min	0.30	0.15	2.42*	0.12***
				
FEMALES				
Constant	3.83		5.73***	
Jogging with others	0.40	0.21	3.21**	
Swimming, diving	-0.30	-0.16	-2.51*	
Hiking	0.27	0.14	2.22*	
Waterskiing	0.30	0.13	2.03*	0.10***

Stepwise regression analysis, only activities reported by at least 10% of the respondents were included.

* *p *< .05, ** *p *< .01, *** *p *< .001

## Discussion

The findings suggest that the transition from adolescence to adulthood is a period of general decline in physical activity, but with the decline levelling off in adulthood. However, the subject-specific analyses revealed a strong heterogeneity in change.

One of the major contributions of the present paper was the assessment of heterogeneity in change of activity from ages 13 to 23. As suggested by most previous longitudinal studies, the average rate of participation in global leisure-time physical activity decreased among both sexes in the adolescent period from 13 to 18 years [2,15,22,23,25-29]. Although at a group level males and females tended to show a pattern of decline in physical activity, there were substantial individual differences in change, particularly among males. Males in the 10^th ^percentile of change would reduce their weekly frequency of global leisure-time physical activity by less than 1 unit across the period, whereas males in the 90^th ^percentile of change would reduce their level of global leisure-time physical activity by about three units per week, and their level of recreational activity by two units per week. This diversity might suggest a need for a more targeted approach, focusing on subgroups of individuals.

The observed heterogeneity has methodological implications. In previous studies, the tracking model has been among the most influential approaches to studying activity across time. The strong heterogeneity in change observed in this study indicates that the tracking model may not be a good representation of activity from adolescence to adulthood. The tracking model assumes that individuals retain their relative positions, but in the presence of strong average and individual change, a focus on relative position might not reveal the most important temporal features. As suggested in the present study, although they show a qualitatively uniform pattern of decline in activity, adolescents change by different amounts, rendering retaining relative positions across time highly unlikely.

Previous studies have indicated a higher level of activity in males compared with females. The growth curve analysis revealed a reduction of sex differences in activity across the adolescent period. While males had a substantially higher level of activity than females at the start of the adolescent period, there was essentially no group difference at age 23. These findings are in line with two other long-term longitudinal studies [4,13], which demonstrated a significant decrease in habitual physical activity during adolescence and early adulthood, with a significantly greater decrease among males. In the Amsterdam Longitudinal Growth and Health Study [13], the reduction of physical activity between the ages of 13 and 27 was 42% in male subjects and 17% in females. Although they did not provide information about causal mechanisms, these findings might indicate a stronger incentive for taking up physical activity in low-activity females. These findings may also suggest a change in the pattern of adult sex differences. Studies before the year 2000 typically reported higher levels of physical activity among adult males, while the present study confirms the findings from other recent studies [4,13], suggesting that females are becoming more physically active during their leisure time. The exponential growth of fitness centres in the Western world during the past 10 years may partly explain this change, as suggested by the finding that almost half of the young women in this study reported exercising at least once a week at a fitness centre.

Males with a low level of activity as 13-year-olds stand out as a particular risk group for low activity at age 23. The analysis conditioning on baseline status indicated that males with a low level of activity at baseline were also likely to show a low level of activity ten years later. In contrast, females with a low level of activity at baseline increased their level of activity throughout the period. This indicates that males with low adolescent activity might be an important target for reinforcement of activity.

To some extent, the relatively stable participation in recreational outdoor activity may be explained by cultural characteristics. In Norway, most adolescents have daily access to nature, recreation parks and outdoor activity areas. It is common to spend leisure time doing outdoor activities, especially during weekends and holidays. Further, many political documents have in recent years more frequently used the term "outdoor activity" in their statements [30]. The increased consciousness and interest in recreational outdoor activity may be another explanation for stability shown in levels of recreational outdoor activity during adolescence and young adulthood.

Participation in most of the specific types of activities showed a significant decline from age 15 to 23, probably because the opportunities to participate in some of these activities are very different for 23-year-olds compared with 15-year-olds. Many of these activities, especially those depending on a certain degree of social structure and organization, are not offered to adults to the same extent as to children and adolescents. In support of Sallis et al.'s [18] suggestion that physical activities that can be performed without a team may more easily carry over from adolescence to adulthood, the findings of this study indicated that individual "adult-like" activities such as walking, hiking and hard work such as gardening and strenuous household chores did not decline.

In line with the findings from the study of the Northern Finland 1966 birth cohort [15], the present results suggest a significant tracking of several individual sports, notably jogging alone and cycling, as well as recreational activities such as skiing and hiking. However, adolescent participation in ball games, which usually take place in a structured context, was also significant. Respondents who participated in ball games at least once weekly at age 15 had increased odds of playing such games at age 23. Thus, certain types of organized youth sports seem to carry over into adulthood.

The individual and team activities mentioned above were also significantly related to global leisure-time physical activity at age 23, suggesting that they may transfer into other types of adult physical activity, but the associations were weak. Thus, the findings suggest that participation in particular sports during adolescence influences global physical activity level at a later stage to a very small extent, and that it may be difficult to identify which types of activities are more important. One reason for this difficulty is that at age 15, adolescents seemed to be involved in several activities simultaneously, thus making it difficult to identify the effect of one single activity. In line with this finding, being involved in several activities at age 15 was found to be moderately correlated to leisure-time physical activity at age 23. It is likely that the specific types of physical activities included in the present study can be characterized as frequent and repeated behaviour in a structured environmental context. Following the assumptions of Verplanken & Melkevik [17] regarding physical activity forming a habit, such activities may then offer good opportunities for establishing lifelong involvement in physical activity, independent of the specific type of activity.

The present study has several limitations. First, the measurements of physical activity were based on self-report data. As pointed out by Shepherd [31], such measurements generally have limited reliability and validity. The inaccuracy of the self-report measures applied in this study may have resulted in lower observed associations between physical activity at early and late stages than if the measures were more accurate. Although self-reported measures may be poor indicators of actual energy expenditure, they may be regarded as good indicators of how active people consider themselves to be. Thus, even if their reporting of frequency (and intensity) of activity may be inaccurate in terms of energy expenditure, self report may still differentiate between different groups of people with regard to broad levels of physical activity. Moreover, the test-retest reliability of the measure of general leisure-time physical activity in this study was found to be acceptable, suggesting that the findings can be trusted.

Self-report measures of physical activity generally seem to overestimate levels of physical activity as directly measured by accelerometers. To the degree that such overestimation is constant across ages, it is not likely to affect the present findings. But if this type of measurement error, as well as other types of measurement error, varies over time as children develop at different rates, the observed changes in physical activity over time could be explained by changes in the ability to fill in self report questionnaires rather than actual behaviour change. Moreover, social desirability may have affected the reporting of physical activity more in young adulthood than in early adolescence, resulting in an overestimation at a later age compared to a younger age. Thus, the decrease in physical activity levels may have been larger than the findings suggest.

Regarding validity, self-report measures are not able to accurately capture all levels of activity and movement. However, self-report measures are able to capture an individual's perception of type of activity (e.g. recreational, exercise, sport) [31]. As one of the main purposes of this study was to study stability and tracking of specific activities, self report measures may actually be consider more relevant than direct measures.

A number of factors may confound the findings, such as parental socioeconomic position. However, previous analyses of these data have suggested that the sample is relatively homogenous in terms of socioeconomic status, and that parental socioeconomic status was not related to changes in health behaviours from ages 13 to 21 [32].

The conclusions from this study may also be limited due to a possible selection bias in the sample. More males dropped out of the study, while females dropping out of the study had a higher baseline vigorous physical activity, which may imply that the findings are less representative for females with a high level of physical activity in early adolescence. The sample of respondents who participated in the last data collection may to some extent have been self-selected, and may differ from the initial sample in terms of factors that may affect tracking of physical activity, for example by resulting in estimates indicating higher tracking than without such a potential bias. Thorough drop-out analyses were conducted on all study variables, suggesting small and mainly insignificant differences between those who dropped out of the study and those who stayed on, suggesting negligible selection bias.

The main strength of this study is the longitudinal design from early adolescence to young adulthood, multiple time points, a relatively large sample with a relatively low drop-out rate, comprehensive self-report measures of physical activity and consistent use of instruments.

## Conclusion

There is considerable individual variation in the degree to which frequency of leisure-time physical activity during adolescence is maintained into adulthood. The degree of maintenance differs across activity types and activity domains. This diversity might suggest a need to target subgroups of individuals. The observed heterogeneity in change highlights the limitation of previous approaches to analyzing physical levels over time, suggesting that growth curve analysis or similar approaches should be used in future research.

There was a significant decrease in most types of activity between the ages of 13 and 23, and the decrease was greater among males than females. The group of inactive youth may be considered as a high risk group, and the findings suggest that adolescent males who are inactive early seem likely to continue to be inactive later. Intervention studies could be initiated to organize physical activity for this group. The aim would be to motivate young inactive men to change their physical activity patterns into more physically active ways of living on their own conditions.

The findings indicate low associations between participation in specific types of activities during adolescence and global leisure-time physical activity in young adulthood, while participation in several adolescent physical activities simultaneously was moderately related to later activity. Thus, it seems that such activities may offer good opportunities for establishing lifelong involvement in physical activity, independent of the specific type of activity. These findings imply that it is important to promote the availability of various types of activities and facilities in youth sport, and perhaps also to avoid too much specialization in particular sports at a young age.

## Competing interests

The authors declare that they have no competing interests.

## Authors' contributions

All three authors contributed to conceptualizing and writing the manuscript, TT carried out the growth curve analysis.

## References

[B1] Malina RM (2001). Tracking of physical activity across the lifespan. Research Digest President's Council on Physical Fitness and Sports Washington DC Series 3.

[B2] Aaron DJ, Storti KL, Robertson RJ, Kriska AM, LaPorte RE (2002). Longitudinal study of the number and choice of leisure time physical activities from mid to late adolescence. Implications for school curricula and community recreation programs. Arch Pediatr Adolesc Med.

[B3] Trost SG, Owen N, Bauman AE, Sallis JF, Brown W (2002). Correlates of adults'participation in physical activity: review and update. Med Sci Sports Exerc.

[B4] Telama R, Yang X, Viikari J, Välimäki I, Wanne O, Raitakari O (2005). Physical activity from childhood to adulthood – a 21 – year tracking study. Am J Prev Med.

[B5] McMurray RG, Harrell JS, Bangdiwala Si, Hu J (2003). Tracking of physical activity and aerobic power from childhood through adolescence. Med Sci Sports Exerc.

[B6] Trudeau F, Laurencelle L, Shephard RJ (2004). Tracking of physical activity from childhood to adulthood. Med Sci Sports Exerc.

[B7] Malina RM (1996). Tracking of physical activity and physical fitness across the lifespan. Res Q Exerc Sport.

[B8] Anderssen N, Wold B, Torsheim T (2005). Tracking of exercise in adolescence. Res Q Exerc Sport.

[B9] Beunen GP, Lefevre J, Philippaerts RM, Delvaux K, Thomis M, Claessens AL, Vanreusel B (2004). Adolescent correlates of adult physical activity: A 26-year Follow up. Med Sci Sports Exerc.

[B10] Kristensen PL, Møller NC, Korsholm L, Wedderkopp NN, Andersen LB, Froberg K (2008). Tracking of objectively measured physical activity from childhood to adolescence: The European Youth Heart study. Scand J Med Sci Sports.

[B11] Matton L, Thomis M, Wijndaele K (2006). Tracking of physical fitness and physical activity from youth to adulthood in females. Med Sci Sports Exerc.

[B12] Perkins DF, Jacobs JE, Barber BL, Eccles JS (2004). Childhood and adolescent sports participation as predictors of participation in sports and physical fitness activities during young adulthood. Youth & Soc.

[B13] van Mechelen WT, Twisk JWR, Post GB, Snel J, Kemper HCG (2000). Physical activity of young people: The Amsterdam Longitudinal Growth and Health Study. Med Sci Sports Exerc.

[B14] Perry CL, Stone EJ, Parcel GS, Ellison RC, Nader PR, Webber LS, Luepker RW (1990). School-based cardiovascular health promotion – the child and adolescent trial for cardiovascular health (CATCH). J School Health.

[B15] Tammelin T, Näyhä S, Hills AP, Järvelin MR (2003). Adolescent participation in sports and adult physical activity. Am J Prev Med.

[B16] TRP Special Report (282) (2005). Does the built Environment Influence Physical Activity – Examining the evidence.

[B17] Verplanken B, Melkevik O (2008). Predicting Habit: The case of physical exercise. Psyc Sport & Exerc.

[B18] Sallis J, Zacarian J, Howell M, Hofstetter R (1996). Ethnic, socioeconomic, and sex differences in physical activity among adolescents. J Clin Epidem.

[B19] Telama R, Yang X, Hirvenssalo M, Raitakari O (2006). Participation in organized youth sport as a predictor of adult physical activity: A 21-year longitudinal Study. Pediatr Exerc Sci.

[B20] Samdal O, Tynjala J, Roberts C, Sallis JF, Villberg J, Wold B (2007). Trends in physical activity and Tv watching of adolescents from 1986 to 2002 in seven European Countries. Eur J Public Health.

[B21] Booth ML, Okeley AD, Chey T, Bauman A (2002). The reliability and validity of the adolescent physical activity recall questionnaire. Medicine and Science in Sports and Exercise.

[B22] Aarnio M, Winter T, Peitonen J, Kujala EM, Kaprio J (2002). Stability of leisure-time physical activity during adolescence – a longitudinal study among 16-. 17-, and 18-year-old Finnish youth. Scand J Med Sci Sports.

[B23] Kemper HCG, Twisk JWR, Koppes LLJ, van Mechelen W, Post GB (2001). A 15-year physical activity pattern is positively related to aerobic fitness in young males and females (13–27 years)'. Eur J Appl Physiol.

[B24] Singer JD, Willett JB (2003). Applied longitudinal data analysis: Modeling change and event occurrence.

[B25] West P, Reeder AI, Milne BJ, Poulton R (2002). Worlds apart: a comparison between physical activities among youth in Glasgow, Scotland and Dunedin, New Zealand. Soc Sci and Med.

[B26] Kristjansdottir G, Viljalmsson R (2001). Sociodemographic differences in patterns of sedate and physically active behavior in older children and adolescents. Acta Pædiatr.

[B27] Telama R, Leskinen E, Yang X (1996). Stability of habitual physical activity and sport participation: a longitudinal tracking study. Scand J Medic Sci Sports.

[B28] Telama R, Yang X, Laakso L, Viikarii J (1997). Physical Activity in Childhood and Adolescence as Predictor of Physical Activity in Young Adulthood. Am J Prev Med.

[B29] Telama R, Laakso L, Yang X (1994). Physical activity and participation in sports of young people in Finland. Scand J Med Sci Sports.

[B30] White Paper number 16 (2002). 'Prescription for a healthy Norway.

[B31] Shephard RJ (2003). Limits to the measurements of habitual physical activity by questionnaires. Brit J Sports Med.

[B32] Friestad C, Klepp KI (2006). Socioeconomic status and health behaviour patterns through adolescence: Results from a prospective cohort study in Norway. Eur J Pub Health.

